# Baseline high-sensitivity C-reactive protein predicts the risk of incident ankylosing spondylitis: Results of a community-based prospective study

**DOI:** 10.1371/journal.pone.0211946

**Published:** 2019-02-15

**Authors:** Jinmei Su, Liufu Cui, Wenhao Yang, Huijing Shi, Cheng Jin, Rong Shu, Hongfen Li, Xiaofeng Zeng, Shouling Wu, Xiang Gao

**Affiliations:** 1 Department of Rheumatology, Peking Union Medical College Hospital, Peking Union Medical College, Chinese Academy of Medical Sciences, Beijing, China; 2 Department of Rheumatology, Kailuan General Hospital, Tangshan, China; 3 Department of Cardiology, Kailuan General Hospital, Tangshan, China; 4 Department of Nutritional Science, Pennsylvania State University, University Park, Pennsylvania, United States of America; 5 Department of Central Laboratory, Kailuan General Hospital, Tangshan, China; Beijing Key Laboratory of Diabetes Prevention and Research, CHINA

## Abstract

**Background:**

A hospitalized-based cohort study suggested that elevated C-reactive protein (CRP) levels are associated with radiographic sacroiliitis progression in ankylosing spondylitis (AS) patients. However, data from community-based populations are limited.

**Objective:**

We sought to determine the association between elevated CRP levels and AS diagnosis in a prospective community-based study of 129,681 Chinese adults over a follow-up period of 8 years.

**Methods:**

We measured the plasma CRP concentration at baseline and every 2 years thereafter with the high-sensitivity (hs)-CRP test. Incident AS cases were confirmed on the basis of modified New York diagnostic criteria after review of medical records. We used Cox proportional-hazard models to calculate hazard ratios (HRs) and 95% confidence intervals (95% CIs) for AS on the basis of hs-CRP concentrations, adjusting for age, sex, education, income, cigarette smoking, alcohol intake, physical activity, body mass index, blood-pressure status, blood glucose status, total cholesterol, history of cardiovascular disease, and use of antihypertensives, lipid-lowering agents, and aspirin.

**Results:**

During 1,033,609 person-years (average 7.97 ± 1.36 years per person) of follow-up, we identified 55 incident AS cases. Baseline hs-CRP was positively associated with the risk of future AS. Compared with hs-CRP <1 mg/L, the HR was 1.28 (95% CI 0.54–3.08) for hs-CRP of 1.00–2.99 mg/L, 4.71 (95% CI 2.26–9.81) for hs-CRP of 3.00–9.99 mg/L, and 19.8 (95% CI 9.6–40.9) for hs-CRP ≥10.00 mg/L (*P*-trend <0.001) after adjustment for potential confounders. We found similar results after excluding AS cases that occurred in the first 2 years of follow-up, and using the cumulative average hs-CRP concentration as a predictor.

**Conclusion:**

This is the first study in a community-based cohort to demonstrate that CRP plasma concentrations predict the risk of future AS, thus providing a test that is easy to routinely perform in the clinic to assess for AS risk.

## Introduction

Ankylosing spondylitis (AS) is a chronic, progressive, inflammatory rheumatic disease that mainly affects the axial skeleton, large peripheral joints, and entheses [1], with a prevalence between 0.1% and 1.4% globally. AS is typified by sacroiliitis and ankyloses of the spine. Structural damage can lead to impaired spinal mobility, decreased participation in daily activities and severely reduced health-related quality of life [2]. On average, it takes 6 to 8 years from the onset of back pain until a clinician establishes a definite diagnosis of AS. Thus, it is very important to predict the risk of developing AS. However, to date, only limited risk factors for AS have been identified, such as human leukocyte antigen B27, family history of AS, male sex and frequent gastrointestinal infections [3].

Radiographic sacroiliitis is considered a hallmark of AS. Inflammation in axial spondyloarthritis starts in the sacroiliac joints. Inflammation of the sacroiliac joints and the spine eventually may lead to bony ankylosis. Therefore, inflammation is a paramount factor in the radiographic assessment of AS progression. A recent study demonstrated that inflammation may predict radiographic progression of AS. In the German Spondyloarthritis Inception Cohort (GESPIC), elevated levels of CRP, a marker of systemic inflammation, were a strong positive predictor of sacroiliitis progression in AS patients [4]. However, it remains unknown whether high CRP concentrations precede the onset of AS in the general population.

In this context, we hypothesized that elevated CRP levels were associated with the incidence of AS. Thus, we tested this hypothesis in a prospective community-based study of approximately 130,000 Chinese adults over a follow-up period of approximately 8 years. Furthermore, we conducted several sensitivity analyses to test our results, including a 2-year-lag analysis, using the average hs-CRP concentration as an independent variable and an analysis restricted to never-smokers.

## Materials and methods

### Study design and population

The Kailuan Study, a prospective cohort study based in the Kailuan community in Tangshan city in northern China, was designed to investigate risk factors for common chronic diseases [5,6]. In 2006, all 155,418 employees of the Kailuan Group ≥18 years old (including retired individuals) were invited to participate in questionnaire assessments and clinical and laboratory examinations conducted in 11 hospitals responsible for the health-care of this community. A total of 101,510 participants comprising Kailuan Study I completed questionnaires and clinical examinations between 2006 and 2007. In 2008–2010, 35,856 adults who did not participate in Kailuan Study I were enrolled in Kailuan Study II (S1 Fig). In both studies, the same questionnaires and clinical and laboratory examinations were administered every 2 years, to update the participants’ health and lifestyle status data until the end of follow-up on December 31, 2015.

The 137,349 participants in Kailuan studies I and II were considered for their eligibility for inclusion in the current study, resulting in exclusion of 7,645 participants with missing information relating to hs-CRP, and 95 participants who had AS at baseline, and leaving 129,681 participants for inclusion. 121,656 participants were remained at the end of study on December 31, 2015 with 8,025 lost during follow-up (S1 Fig).

The protocol for this study was in accordance with the guidelines of the Helsinki Declaration and was approved by the Ethics Committee of the Kailuan Medical Group, Kailuan Company. Written informed consent was obtained from all participants. Because of confidentiality issues, the authors cannot post the data directly. However, de-identified data are available to researchers upon request by contacting Dr Shouling Wu.

### Incidence of AS

Incident AS cases were identified by searching the Municipal Social Insurance Institution and the Hospital Discharge Register of the 11 hospitals (with an ICD-10 search code of M45 for AS). Three rheumatologists (Drs Wenhao Yang, Huijing Shi and Jinmei Su) reviewed all medical records and confirmed the diagnosis of AS on the basis of fulfilment of the modified New York classification criteria [7].

### Assessment of hs-CRP concentrations

Fasting (8–12 h) venous blood samples were drawn during each study visit and analysed at the Central Laboratory of Kailuan General Hospital on the same day [6]. Plasma hs-CRP concentrations were measured with a high-sensitivity particle-enhanced immunonephelometry assay (Cias Latex CRP-H, Kanto Chemical, Tokyo, Japan). The lower limit of detection was 0.1 mg/L. The intra-assay coefficient of variation was 6.53%, and the inter-assay coefficient of variation was 4.78%. The concentrations of hs-CRP were measured at baseline and in the subsequent biennial survey for both study cohorts.

### Assessment of covariates

Information on demographic variables (including age, sex, education, income, smoking status, alcohol intake, and physical activity) was collected via questionnaires administered by the research doctors at the baseline interview. Smoking status was divided into three categories (current, past, or never); alcohol intake was divided into five categories (never, past, light (women: 0–0.4 servings per day, men: 0–0.9 servings per day, where a serving equates to 15 g of alcohol), moderate (women: 0.5–1.0 servings per day, men: 1–2 servings per day), or heavy (women: >1.0 servings per day, men: >2 servings per day)) [8]; and physical activity was divided into three categories (never, 1–2 times/week, or 3+ times/week) [9].

Bodyweight, height, and blood pressure were measured during the baseline interview, and body mass index (BMI) was calculated using the weight in kilograms divided by the square of the height in metres. Blood pressure was the average of at least two readings taken at rest. Standard protocols were used for all measurements. The categories for blood pressure levels were ‘normal’ (systolic blood pressure <120 mmHg and diastolic blood pressure <80 mmHg), ‘prehypertension’ (systolic blood pressure 120–139 mmHg or diastolic blood pressure 80–89 mmHg) and ‘hypertension’ (systolic blood pressure ≥140 mmHg or diastolic blood pressure ≥90 mmHg, or a history of physician-diagnosed hypertension or the use of antihypertensive drugs) [10]. ‘Diabetes’ was diagnosed as fasting blood glucose ≥126 mg/dL, a history of physician-diagnosed diabetes or taking glucose-lowering medication; ‘impaired fasting glucose’ was diagnosed as fasting blood glucose 100–125 mg/dL; and ‘normal’ was diagnosed as fasting blood glucose <100 mg/dL [11].

Concentrations of total cholesterol and triglyceride, high-density lipoprotein cholesterol (HDL-C), and low-density lipoprotein cholesterol (LDL-C) were measured with an auto-analyser (Hitachi 747; Hitachi, Tokyo, Japan) as described previously [6,8].

### Statistical analyses

Statistical analyses were performed using SAS software, version 9.3 (SAS Institute, Cary, NC, USA). Demographic information and baseline characteristics of the participants were summarized and compared in relation to the concentrations of hs-CRP at baseline. Continuous variables are presented as the mean ± standard deviation, and discrete variables are presented as counts and percentages. *P*-values were two-tailed, and values <0.05 were considered statistically significant.

The participants were divided into four categories on the basis of the baseline concentrations of hs-CRP (<1.0 mg/L, 1.0–3.0 mg/L, >3.0 mg/L to <10.0 mg/L, and ≥10.0 mg/L) [6,12]. The person-time of follow-up for each participant was determined from the end date of the Kailuan Study I or Study II survey to either the date of AS onset or the end of follow-up (December 31, 2015), whichever came first. Cox proportional-hazards regression was used for calculation of hazard ratios (HRs) and 95% confidence intervals (95% CIs) for AS risk in relation to hs-CRP status. Logistic regression was also performed with penalized maximum-likelihood estimation, because of the small number of incident AS cases. Three multivariate proportional hazards models were fitted, to account for potential confounders. Model 1 adjusted for age and sex. Model 2 also adjusted for education, average monthly income of each family member, smoking, alcohol intake, and physical activity. Model 3 further adjusted for BMI, blood-pressure status, blood glucose status, total cholesterol, history of cardiovascular disease, and the use of antihypertensives, lipid-lowering agents, and aspirin. Categories for each of these variables were as shown in Table 1.

**Table 1:** Baseline characteristics of 129,681 study participants according to hs-CRP concentrations at baseline.

Characteristic	Plasma concentration of hs-CRP (mg/L)				*P*
	<1.00	1.00–2.99	3.00–9.99	≥10.00	
*n*	66,228	36,345	21,737	5,371	
hs-CRP (mg/L), median (Q1,Q3)	0.40 (0.20,0.63)	1.60(1.21,2.10)	4.90(3.70,6.80)	14.8(11.6,23.1)	
Age (years), mean ± SD	49.3 ± 12.4	51.5 ± 13.0	54.4 ± 12.9	55.4 ± 13.5	<0.001
Men, *n* (%)	53,557 (80.9)	29,725 (81.8)	17,294 (79.6)	4,356 (81.1)	0.01
BMI (kg/m^2^), mean ± SD	24.4 ± 3.3	25.6 ± 3.5	25.7 ± 3.7	25.4 ± 3.9	<0.001
Education, *n* (%)					
Illiteracy or elementary school	5,749 (8.7)	3,903 (10.7)	2,896 (13.3)	769 (14.3)	<0.001
Middle school	54,375 (82.1)	28,918 (79.6)	17,034 (78.4)	4,145 (77.2)	<0.001
College/university	6,026 (9.1)	3,440 (9.5)	1,640 (7.5)	392 (7.3)	<0.001
Average income, *n* (%)					
<¥500/month	16,378 (24.7)	7,874 (21.7)	3,908 (18.0)	1,023 (19.0)	<0.001
¥500–2,999/month	42,045 (63.5)	22,531 (62.0)	13,109 (60.3)	3,213 (59.8)	<0.001
≥¥3,000/month	4,380 (6.6)	2,686 (7.4)	2,581 (11.9)	585 (10.9)	<0.001
Smoking status, *n* (%)					
Current	3,338 (5.0)	2,157 (5.9)	1,301 (6.0)	338 (6.3)	<0.001
Past	23,844 (36.0)	13,497 (37.1)	6,991 (32.2)	1,652 (30.8)	<0.001
Never	38,989 (58.9)	20,621 (56.7)	13,291 (61.1)	3,318 (61.8)	<0.001
Alcohol intake, *n* (%)					
Never	38,973 (58.8)	20,246 (55.7)	13,281 (61.1)	3,277 (61.0)	<0.001
Light^a^	8,175 (12.3)	4,876 (13.4)	2,337 (10.8)	500 (9.3)	<0.001
Moderate^b^	2,885 (4.4)	1,613 (4.4)	846 (3.9)	216 (4.0)	0.013
Heavy^c^	9,486 (14.3)	4,564 (12.6)	2,156 (9.9)	523 (9.7)	<0.001
Past	1,905 (2.9)	1,179 (3.2)	690 (3.2)	210 (3.9)	<0.001
Physical activity, *n* (%)					
Never	8,476 (12.8)	5,100 (14.0)	2,779 (12.8)	598 (11.1)	0.41
1–2 times/week	47,633 (71.9)	24,479 (67.4)	15,512 (71.4)	3,935 (73.3)	0.003
3+ times/week	10,050 (15.2)	6,691 (18.4)	3,271 (15.0)	764 (14.2)	0.179
TC (mmol/L), mean ± SD	4.88 ± 1.12	5.00 ± 1.11	4.97 ± 1.11	4.90 ± 1.12	<0.001
TG (mmol/L), mean ± SD	1.56 ± 1.31	1.78 ± 1.47	1.78 ± 1.47	1.65 ± 1.37	<0.001
LDL-C (mmol/L), mean ± SD	2.43 ± 0.78	2.52 ± 0.85	2.34 ± 1.17	2.10 ± 1.28	<0.001
HDL-C (mmol/L), mean ± SD	1.54 ± 0.41	1.49 ± 0.41	1.51 ± 0.43	1.56 ± 0.52	<0.001
Blood-pressure status, *n* (%)					
Normal	26,106 (39.4)	11,539 (31.8)	6,234 (28.7)	1,601 (29.8)	<0.001
Prehypertension	16,637 (25.1)	8,498 (23.4)	4,966 (22.9)	1,138 (21.2)	<0.001
Hypertension	23,485 (35.5)	16,308 (44.9)	10,537 (48.5)	2,632 (49.0)	<0.001
Blood glucose status, *n* (%)					
Normal	48,182 (72.8)	23,891 (65.7)	14,337 (66.0)	3,524 (65.6)	<0.001
Prediabetes	13,153 (19.9)	8,298 (22.8)	4,402 (20.3)	1,060 (19.7)	0.004
Diabetes	4,893 (7.4)	4,156 (11.4)	2,998 (13.8)	787 (14.7)	<0.001
History of CVD, *n* (%)	2,850 (4.3)	2,248 (6.2)	1,544 (7.1)	450 (8.4)	<0.001
Antihypertensive treatment, *n* (%)	8,265 (12.5)	6,642 (18.3)	4,416 (20.3)	1,110 (20.7)	<0.001
Antihyperglycemic treatment, *n* (%)	2,165 (3.3)	1,824 (5.0)	1,401 (6.5)	391 (7.3)	<0.001
Lipid-lowering treatment, *n* (%)	857 (1.3)	683 (1.9)	527 (2.4)	140 (2.6)	<0.001
Use of aspirin, *n* (%)	821 (1.2)	755 (2.1)	569 (2.6)	164 (3.1)	<0.001

^a^Light: 0.1–0.4 servings per day (women), 0.1–0.9 servings per day (men).

^b^Moderate: 0.5–1.5 servings per day (women), 1–2 servings per day (men).

^c^Heavy: >1.5 servings per day (women), >2 servings per day (men). A serving equates to 15 g of alcohol.

BMI, body mass index; CVD, cardiovascular disease; HDL-C, high-density lipoprotein cholesterol; hs-CRP, high-sensitivity C-reactive protein; LDL-C, low-density lipoprotein cholesterol; TC, total cholesterol; TG, triglycerides.

Three sensitivity analyses were conducted. Because elevated hs-CRP concentrations may have been a consequence of impending AS, a 2-year-lag analysis was conducted by excluding AS events from the first 2 years of follow-up, to reduce potential reverse causality. To take advantage of the repeated assessment of hs-CRP in both studies, another sensitivity analysis was conducted using cumulative average hs-CRP concentrations as an exposure. For example, the average hs-CRP concentrations for the years 2006, 2008 and 2010 were used for prediction of the occurrence of AS after 2010. Finally, because smoking could be associated with new radiographic progression [13,14], one analysis was restricted to never-smokers.

Potential interactions between hs-CRP concentrations and sex and age (<45 years and ≥45 years) were also explored in relation to the risk of AS, adjusted for potential confounders.

## Results

Among the 129,681 individuals who were included in this study, relative to those with lower baseline hs-CRP concentrations, individuals with higher hs-CRP concentrations were more likely to have higher bodyweight and age, to be current smokers, to have lower education, higher plasma concentrations of cholesterol, and lower concentrations of LDL-C. A significantly higher proportion of individuals with higher baseline concentrations of hs-CRP had hypertension, diabetes, or a history of cardiovascular diseases or used antihypertensive treatment, antihyperglycemic treatment, lipid-lowering treatment or aspirin, compared with individuals with lower baseline hs-CRP concentrations (Table 1).

**Fig 1:**
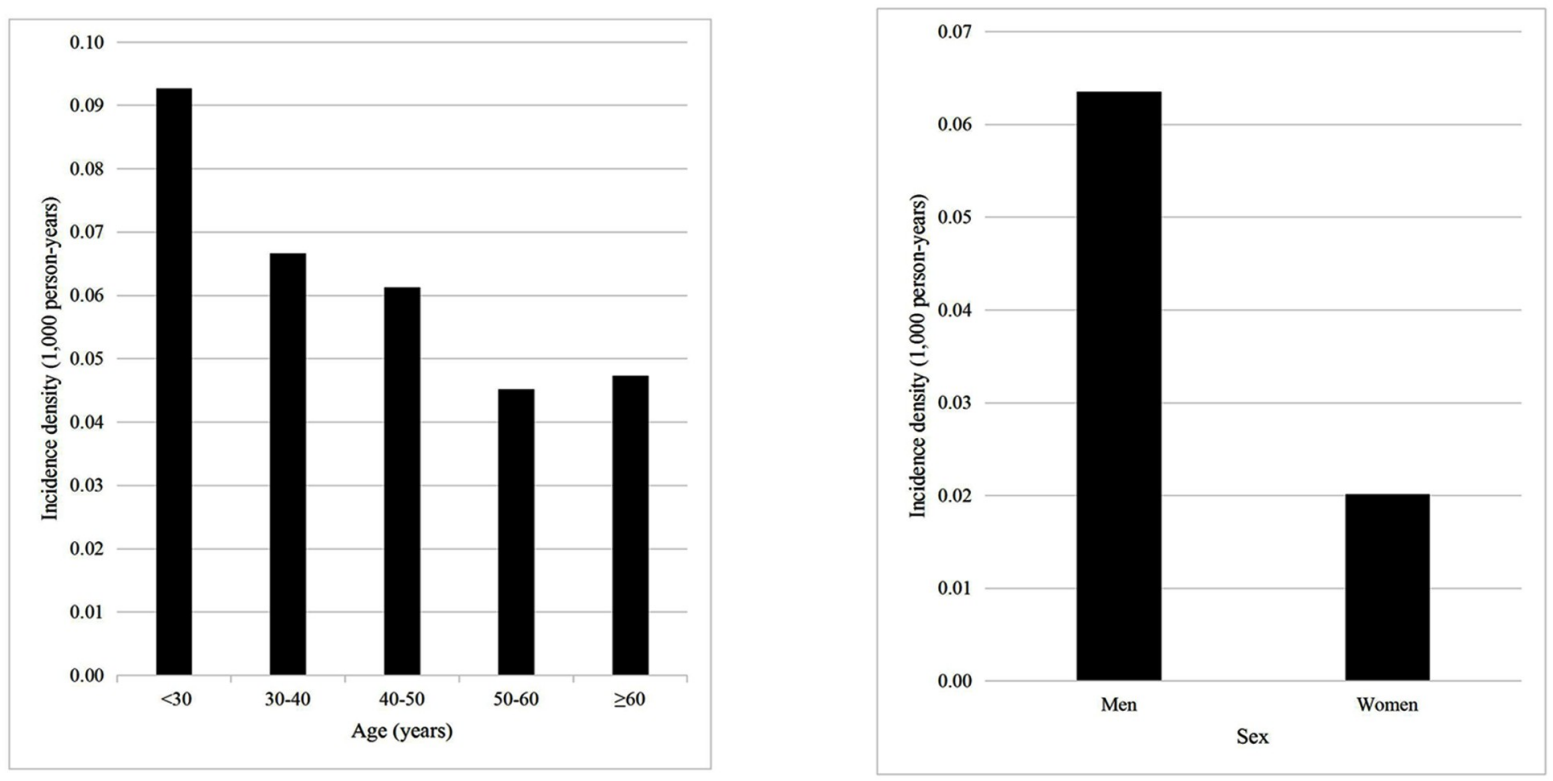
Incidence rates of ankylosing spondylitis (AS) according to age and sex. For the 129,681 individuals included in this study (with a total of 1,033,609 person-years), the incidence density (incidence per 1,000 person-years) was calculated according to age or to sex.

During the 1,033,609 person-years (average 7.97 ± 1.36 years) of follow-up, we documented 55 new AS cases: 14 for baseline hsCRP <1.00 mg/L (an incidence rate of 0.03 per 1,000 person-years), 8 for baseline hsCRP 1.00–2.99 mg/L (0.03 per 1,000 person-years), 16 for baseline hsCRP 3.00–9.99 mg/L (0.09 per 1,000 person-years), and 17 for baseline hsCRP ≥10.0 mg/L (0.39 mg/L). The incidence rates of AS were higher in men than in women and decreased with increasing age (Fig 1). Baseline hs-CRP concentrations were significantly positively associated with future AS risk (*P*-trend <0.001, Table 2). Relative to hs-CRP <1.00 mg/L, HR = 1.28 (95% CI 0.54–3.08) for hs-CRP of 1.00–2.99 mg/L, HR = 4.71 (95% CI 2.26–9.81) for hs-CRP of 3.00–9.99 mg/L, and HR = 19.8 (95% CI 9.6–40.9) for hs-CRP ≥10.0 mg/L (*P*-trend <0.001), after adjusting for potential confounders (multivariate model 2). Exclusion of eight AS cases that occurred in the first 2 years of follow-up, the use of cumulative hs-CRP as an exposure, and exclusion of smokers all generated similar results (Table 2). The same trend was found in analyses of subgroups stratified by sex and by age (Table 3). Significant associations of baseline hs-CRP concentrations with AS incidence were also demonstrated in two multivariate logistic regression analyses (all *P*-trend <0.001, S1 Table).

**Table 2:** Adjusted hazard ratios (HRs) for ankylosing spondylitis according to hs-CRP concentrations at baseline, using different models and sensitivity analyses.

	Plasma concentrations of hs-CRP (mg/L)				
Model	<1.00	1.00–2.99	3.00–9.99	≥10.0	*P*_for trend_
Age- and sex-adjusted model	1.00	1.15 (0.48–2.75)	4.25 (2.06–8.77)	18.2 (8.9–37.3)	<0.001
Multivariate model 1^a^	1.00	1.22 (0.51–2.90)	4.50 (2.17–9.33)	19.2 (9.3–39.4)	<0.001
Multivariate model 2^b^	1.00	1.28 (0.54–3.08)	4.71 (2.26–9.81)	19.8 (9.6–40.9)	<0.001
Sensitivity analyses^b^					
Using cumulative average hs-CRP	1.00	1.48 (0.61–3.58)	7.0 (3.2–15.5)	51.6 (22.9–116.2)	<0.001
2-year-lag analysis	1.00	2.24 (0.72–7.00)	7.8 (2.9–21.1)	29.5 (10.8–80.0)	<0.001
Excluding smokers	1.00	2.22 (0.64–7.79)	4.5 (1.3–16.0)	49.8 (17.3–143.4)	<0.001

hs-CRP, high-sensitivity C-reactive protein. Data presented are hazard ratios relative to <1.00 mg/L hs-CRP, with 95% confidence intervals.

^a^Model adjusted for age, sex, education, average monthly income of each family member, smoking, alcohol intake, and physical activity.

^b^Model included the variables in model 1, and was further adjusted for body mass index, blood-pressure status, blood glucose status, total cholesterol, history of cardiovascular disease, and use of antihypertensives, lipid-lowering agents, and aspirin.

**Table 3:** Incidence of ankylosing spondylitis (AS) according to hs-CRP concentration, with stratification by sex and age.

	Plasma concentrations of hs-CRP (mg/L)				*P*_for trend_	*P*_for interaction_
	<1.00	1.00–2.99	3.00–9.99	≥10.0		
Sex						0.39
Male						
*n*	50,750	27,623	16,384	4,147		
Incidence of AS	11	8	15	17		
Incidence rate^a^	0.03	0.04	0.11	0.50		
HR (95% CI)	1.00	1.62 (0.65–4.06)	5.6 (2.5–12.4)	24.9 (11.5–54.1)	<0.001	
Female						
*n*	12,671	6,620	4,443	1,015		
Incidence of AS	3	0	1	0		
Incidence rate^a^	0.03	0.00	0.03	0.00		
HR (95% CI)	1.00	—	1.4 (0.1–14.0)	—	0.88	
Age						0.23
<45 years						
*n*	24,434	11,711	5,090	1,167		
Incidence of AS	8	1	5	6		
Incidence rate^a^	0.04	0.01	0.14	0.71		
HR (95% CI)	1.00	0.31 (0.04–2.46)	3.7 (1.2–11.5)	18.1 (6.1–53.4)	<0.001	
≥45 years						
*n*	41,794	24,634	16,647	4,204		
Incidence of AS	6	7	11	11		
Incidence rate^a^	0.02	0.04	0.08	0.32		
HR (95% CI)	1.00	2.35 (0.79–7.05)	5.5 (2.0–15.2)	20.6 (7.5–56.4)	<0.001	

HR, hazard ratio; CI, confidence interval; hs-CRP, high-sensitivity C-reactive protein. The model was adjusted for age, sex, education, average monthly income of each family member, smoking, alcohol intake, physical activity, body mass index, blood-pressure status, blood glucose status, total cholesterol, history of cardiovascular disease, and use of antihypertensives, lipid-lowering agents, and aspirin.

^a^Incidence rate per 1,000 person-years.

## Discussion

In this large-scale, community-based, prospective study, baseline plasma concentrations of hs-CRP were predictive of the future risk of AS over an average follow-up period of 8 years. The association was independent of lifestyle factors and medical history, including smoking status, alcohol intake, physical activity, obesity, blood pressure, and blood concentrations of glucose and total cholesterol. A similar significant association was observed in several sensitivity analyses and sub-group analyses.

Our results suggest that active systemic inflammation plays an important role in the stage before established AS in a community population. To our knowledge, no similar data from previous prospective, community-based studies are available. However, our results are consistent with previous studies conducted in the hospital-based AS cohort that demonstrated that inflammation, as assessed by CRP plasma concentration, was associated with radiographic progression of both the sacroiliac joint and the spine. In the GESPIC, elevated baseline CRP was identified as a strong predictor (OR 4.7 in a multivariable model) of the radiographic sacroiliitis progression of non-radiographic axial spondyloarthritis to AS after 2 years of follow-up [4]. Furthermore, elevated time-averaged CRP levels (>6 mg/L) relative to levels ≤6 mg/L were significantly and independently associated with the formation of new syndesmophytes and radiographic progression (defined as an increase of two or more modified Stoke Ankylosing Spondylitis Spine Score units after 2 years) in the spine in AS [13]. However, in the Outcome Assessments in Ankylosing Spondylitis International Study cohort, elevated CRP at baseline in patients with AS did not emerge as an independent predictor for the development of new syndesmophytes and/or bridges in the spine at 4 years [15]. These discordant results potentially resulted from the different durations of the disease in these cohorts [13].

Although the mechanism of AS remains unknown, some evidence indicates that inflammation plays an important role in the initiation and progression of AS. First, the traditional pathophysiological hypothesis proposes that gastrointestinal or urogenital infections trigger spondyloarthritis [16]. These infections may be a source of chronic inflammation, as detected by elevated CRP levels. Second, two additional hypotheses propose an autoinflammatory rather than autoimmune origin. Bacterial or mechanical stress induces cytokine production in inflammatory cells in response to a range of innate immune stimuli, and inflammation occurs at sites of bacterial or mechanical stress [17]. Third, histological studies show that inflammatory changes in the enthesis are considered a characteristic finding in AS [18]. In addition, magnetic resonance imaging shows inflammation in the sacroiliac joint and the spine during the acute phase. Finally, anti-inflammatory treatment with nonsteroidal anti-inflammatory drugs [19,20] and tumor necrosis factor α inhibitors [21,22,23] are effective in AS patients. Our study extends the findings of previous studies and provides direct evidence that inflammation could be considered an early feature and/or risk factor for AS.

Our study had a number of potential limitations. First, the Kailuan study, from which our cohort was drawn, was not a nationwide study, and a large proportion of the participants were manual workers. Therefore, our findings may not be directly applicable to another population with a different socioeconomic-status profile. The relationship between baseline hs-CRP and the incidence of AS should therefore be investigated in other prospective cohorts with different backgrounds of socioeconomic status and culture. Second, although we conducted a 2-year lag analysis, the possibility of reverse causality cannot be totally excluded because AS has a long pre-clinical stage. Third, high levels of CRP can occur in many acute conditions (such as infections and allergic reactions) and chronic conditions (such as cardiovascular disease). Although we could not exclude all these conditions, we conducted a sensitivity analysis using cumulative average CRP concentrations as an exposure to reduce any bias from acute disease, and we used a model that included variables that may affect CRP concentrations (such as history of cardiovascular diseases) to reduce bias from chronic disease. Fourth, we did not measure other markers of inflammation, such as amyloid A, interleukin 6 or soluble intercellular adhesion molecule type-1, because these measurements were not routinely conducted in our hospital. Therefore, further studies including more markers of inflammation are warranted.

In conclusion, systemic inflammation status, as assessed by CRP plasma concentration, predicts the risk of AS independent of potential confounders. These results provide a test that is easy to preform and may be used widely in various clinics to predict AS to aid in early diagnosis and treatment. Further prospective studies including participants with different cultural backgrounds and analyses of other markers of inflammation are warranted to attempt to replicate and expand on our findings.

## Supporting information

S1 Fig Management and outcome of individuals of Kailuan Study I and Kailuan Study II. AS, ankylosing spondylitis; hs-CRP, high-sensitivity C-reactive protein.
(DOCX)

S1 Table Adjusted odds ratio (ORs) for ankylosing spondylitis, according to Hs-CRP concentrations at baseline. * Model adjusted for age, sex, education, average monthly income of each family member, smoking, alcohol intake, physical activity. ^#^ Model included the variables in model 1 and further adjusted for body mass index, blood pressure status, blood glucose status, and total cholesterol, history of cardiovascular disease and use of antihypertensives, lipid-lowering agents, and aspirin. CI, confidence interval; hs-CRP, high-sensitivity C-reactive protein.
(DOCX)

S1 Dataset (RAR)

## References

[1] Braun J, Bollow M, Remlinger G, Eggens U, Rudwaleit M, Distler A, et al Prevalence of spondylarthropathies in HLA-B27 positive and negative blood donors. Arthritis Rheum. 1998;41: 58–67. 10.1002/1529-0131(199801)41:1<58::AID-ART8>3.0.CO;2-G 9433870

[2] Davis JC, van der Heijde D, Dougados M, Woolley JM. Reductions in health-related quality of life in patients with ankylosing spondylitis and improvements with etanercept therapy. Arthritis Rheum. 2005;53: 494–501. 10.1002/art.21330 16082640

[3] Sieper J, Braun J, Rudwaleit M, Boonen A, Zink A. Ankylosing spondylitis: an overview. Ann Rheum Dis. 2002;61 Suppl 3: iii8–iii18. 12381506 10.1136/ard.61.suppl_3.iii8 PMC1766729

[4] Poddubnyy D, Rudwaleit M, Haibel H, Listing J, Marker-Hermann E, Zeidler H, et al Rates and predictors of radiographic sacroiliitis progression over 2 years in patients with axial spondyloarthritis. Ann Rheum Dis. 2011;70: 1369–1374. 10.1136/ard.2010.145995 21622969

[5] Wang L, Cui L, Wang Y, Vaidya A, Chen S, Zhang C, et al Resting heart rate and the risk of developing impaired fasting glucose and diabetes: the Kailuan prospective study. Int J Epidemiol. 2015;44: 689–699. 10.1093/ije/dyv079 26002923 PMC4553707

[6] Wu Z, Huang Z, Jin W, Rimm EB, Lichtenstein AH, Kris-Etherton PM, et al Peripheral inflammatory biomarkers for myocardial infarction risk: a prospective community-based study. Clin Chem. 2017;63: 663–672. 10.1373/clinchem.2016.260828 28031418

[7] van der Linden S, Valkenburg HA, Cats A. Evaluation of diagnostic criteria for ankylosing spondylitis. A proposal for modification of the New York criteria. Arthritis Rheum. 1984;27: 361–368. 6231933 10.1002/art.1780270401

[8] Huang S, Li J, Shearer GC, Lichtenstein AH, Zheng X, Wu Y, et al Longitudinal study of alcohol consumption and HDL concentrations: a community-based study. Am J Clin Nutr. 2017;105: 905–912. 10.3945/ajcn.116.144832 28251934 PMC5366050

[9] Zhang Q, Zhou Y, Gao X, Wang C, Zhang S, Wang A, et al Ideal cardiovascular health metrics and the risks of ischemic and intracerebral hemorrhagic stroke. Stroke. 2013;44: 2451–2456. 10.1161/STROKEAHA.113.678839 23868276

[10] Chobanian AV, Bakris GL, Black HR, Cushman WC, Green LA, Izzo JL, et al The seventh report of the joint national committee on prevention, detection, evaluation, and treatment of high blood pressure: the JNC 7 report. JAMA. 2003;289: 2560–2572. 10.1001/jama.289.19.2560 12748199

[11] American Diabetes Association. Clinical practice recommendations 2005. Diabetes Care. 2005;28 Suppl 1: S1–S79. 15618109 10.2337/diacare.28.suppl_1.s1

[12] Pearson TA, Mensah GA, Alexander RW, Anderson JL, Cannon RO 3rd, Criqui M, et al Markers of inflammation and cardiovascular disease: application to clinical and public health practice: a statement for healthcare professionals from the centers for disease control and prevention and the American heart association. Circulation. 2003;107: 499–511. 12551878 10.1161/01.cir.0000052939.59093.45

[13] Poddubnyy D, Haibel H, Listing J, Marker-Hermann E, Zeidler H, Braun J, et al Baseline radiographic damage, elevated acute-phase reactant levels, and cigarette smoking status predict spinal radiographic progression in early axial spondylarthritis. Arthritis Rheum. 2012;64: 1388–1398. 10.1002/art.33465 22127957

[14] Villaverde-Garcia V, Cobo-Ibanez T, Candelas-Rodriguez G, Seoane-Mato D, Campo-Fontecha PDD, Guerra M, et al The effect of smoking on clinical and structural damage in patients with axial spondyloarthritis: a systematic literature review. Semin Arthritis Rheum. 2017;46: 569–583. 10.1016/j.semarthrit.2016.11.004 27979416

[15] van Tubergen A, Ramiro S, van der Heijde D, Dougados M, Mielants H, Landewe R. Development of new syndesmophytes and bridges in ankylosing spondylitis and their predictors: a longitudinal study. Ann Rheum Dis. 2012;71: 518–523. 21989544 10.1136/annrheumdis-2011-200411

[16] Costello ME, Ciccia F, Willner D, Warrington N, Robinson PC, Gardiner B, et al Brief Report: Intestinal Dysbiosis in Ankylosing Spondylitis. Arthritis Rheumatol. 2015;67: 686–691. 10.1002/art.38967 25417597

[17] Dougados M, Baeten D. Spondyloarthritis. Lancet 2011;377:2127–2137. 10.1016/S0140-6736(11)60071-8 21684383

[18] Francois RJ, Eulderink F, Bywaters EG. Commented glossary for rheumatic spinal diseases, based on pathology. Ann Rheum Dis. 1995;54: 615–625. 7677436 10.1136/ard.54.8.615 PMC1009954

[19] Song IH, Poddubnyy DA, Rudwaleit M, Sieper J. Benefits and risks of ankylosing spondylitis treatment with nonsteroidal antiinflammatory drugs. Arthritis Rheum. 2008;58: 929–38. 10.1002/art.23275 18383378

[20] Wanders A, van der Heijde D, Landewé R, Béhier JM, Calin A, Olivieri I, et al Nonsteroidal anti-inflammatory drugs reduce radiographic progression in patients with ankylosing spondylitis: a randomized clinical trial. Arthritis Rheum. 2005;52: 1634–36. 15934081 10.1002/art.21054

[21] Braun J, Brandt J, Listing J, Zink A, Alten R, Golder W, et al Treatment of active ankylosing spondylitis with infliximab: a randomised controlled multicentre trial. Lancet. 2002;359: 1187–93. 11955536 10.1016/s0140-6736(02)08215-6

[22] Davis JC Jr, Van Der Heijde D, Braun J, Dougados M, Cush J, Clegg DO, et al Recombinant human tumor necrosis factor receptor (etanercept) for treating ankylosing spondylitis: a randomized, controlled trial. Arthritis Rheum. 2003:48: 3230–36. 10.1002/art.11325 14613288

[23] van der Heijde D, Kivitz A, Schiff MH, Sieper J, Dijkmans BA, Braun J, et al Efficacy and safety of adalimumab in patients with ankylosing spondylitis: results of a multicenter, randomized, double-blind, placebo-controlled trial. Arthritis Rheum. 2006;54: 2136–46. 10.1002/art.21913 16802350

