# Supplementary material for: Baseline high-sensitivity C-reactive protein predicts the risk of incident ankylosing spondylitis: Results of a community-based prospective study
Source: PLoS One. 2019 Feb 15;14(2):e0211946. doi: 10.1371/journal.pone.0211946 (PMC6377123; doi:10.1371/journal.pone.0211946)
Supplement: S1 Fig — AS, ankylosing spondylitis; hs-CRP, high-sensitivity C-reactive protein. (DOCX) [file pone.0211946.s001.docx]

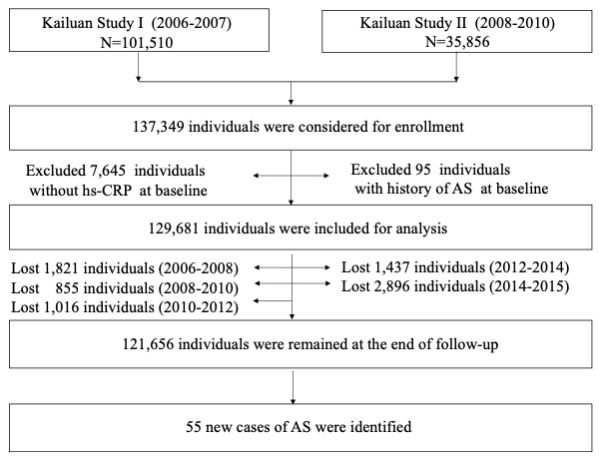


**S1** **Fig. Management and Outcome of Individuals of Kailuan Study I and Kailuan Study II.** AS, ankylosing spondylitis; hs-CRP, high-sensitivity C-reactive protein.
