# Supplementary material for: Baseline high-sensitivity C-reactive protein predicts the risk of incident ankylosing spondylitis: Results of a community-based prospective study
Source: PLoS One. 2019 Feb 15;14(2):e0211946. doi: 10.1371/journal.pone.0211946 (PMC6377123; doi:10.1371/journal.pone.0211946)
Supplement: S1 Table — * Model adjusted for age, sex, education, average monthly income of each family member, smoking, alcohol intake, physical activity. # Model included the variables in model 1 and further adjusted for body mass index, blood pressure status, blood glucose status, and total cholesterol, history of cardiovascular disease and use of antihypertensives, lipid-lowering agents, and aspirin. CI, confidence interval; hs-CRP, high-sensitivity C-reactive protein. (DOCX) [file pone.0211946.s002.docx]

| **S1 Table. Adjusted Odds ratio (ORs) for Ankylosing Spondylitis, According to Hs-CRP Concentrations at Baseline.** | | | | | | | | | | |
| --- | --- | --- | --- | --- | --- | --- | --- | --- | --- | --- |
|  | Plasma concentrations of hs-CRP (mg/L) | | | | | | | | p _for trend_ | |
|  | <1.00 | 1.00-2.99 | | 3.00-9.99 | | ≥10.0 | |  | |  |
| Population | 66,228 | 36,345 | | 21,737 | | 5,371 | |  | |  |
| Number of Case | 14 | 8 | | 16 | | 17 | |  | |  |
|  |  | OR | 95%CI | OR | 95%CI | OR | 95%CI |  | |  |
| Age- and sex-adjusted | 1.00 | 1.14 | 0.49-2.63 | 4.07 | 2.02-8.21 | 17.7 | 8.8-35.5 | <0.001 | |  |
| Multivariate-adjusted model 1^*^ | 1.00 | 1.22 | 0.56-2.64 | 4.37 | 2.27-8.41 | 18.7 | 9.8-35.9 | <0.001 | |  |
| Multivariate-adjusted model 2^$^ | 1.00 | 1.29 | 0.61-2.71 | 4.58 | 2.42-8.64 | 19.4 | 10.3-36.4 | <0.001 | |  |
| ^*^ Model adjusted for age, sex, education, average monthly income of each family member, smoking, alcohol intake, physical activity.  ^#^ Model included the variables in model 1 and further adjusted for body mass index, blood pressure status, blood glucose status, and total cholesterol, history of cardiovascular disease and use of antihypertensives, lipid-lowering agents, and aspirin.  CI, confidence interval; hs-CRP, high-sensitivity C-reactive protein. | | | | | | | | | | |
